# O157:H7 and O104:H4 Vero/Shiga toxin-producing *Escherichia coli *outbreaks: respective role of cattle and humans

**DOI:** 10.1186/1297-9716-43-13

**Published:** 2012-02-13

**Authors:** Denis Piérard, Henri De Greve, Freddy Haesebrouck, Jacques Mainil

**Affiliations:** 1 National Reference Center for Verotoxin/Shiga toxin producing E.coli, Department Microbiology and Infection Control, Universitair Ziekenhuis Brussel, Laarbeeklaan 101, B-1090 Brussels, Belgium; 2 Structural & Molecular Microbiology, Department of Structural Biology, VIB, Brussels, Belgium; 3 Structural Biology Brussels, Vrije Universiteit Brussel, B-1050 Brussels, Belgium; 4 Department of Pathology, Bacteriology and Avian Diseases, Faculty of Veterinary Medicine, Ghent University, B-9820 Merelbeke, Belgium; 5 Bacteriology, Department of Infectious Diseases, Faculty of Veterinary Medicine, University of Liège, B-4000 Liège, Belgium

## Abstract

An enteroaggregative Verotoxin (Vtx)-producing *Escherichia coli *strain of serotype O104:H4 has recently been associated with an outbreak of haemolytic-uremic syndrome and bloody diarrhoea in humans mainly in Germany, but also in 14 other European countries, USA and Canada. This O104:H4 *E. coli *strain has often been described as an enterohaemorrhagic *E. coli *(EHEC), i.e. a Vtx-producing *E. coli *with attaching and effacing properties. Although both EHEC and the German O104:H4 *E. coli *strains indeed produce Vtx, they nevertheless differ in several other virulence traits, as well as in epidemiological characteristics. For instance, the primary sources and vehicles of typical EHEC infections in humans are ruminants, whereas no animal reservoir has been identified for enteroaggregative *E. coli *(EAggEC). The present article is introduced by a brief overview of the main characteristics of Vtx-producing *E. coli *and EAggEC. Thereafter, the O104:H4 *E. coli *outbreak is compared to typical EHEC outbreaks and the virulence factors and host specificity of EHEC and EAggEC are discussed. Finally, a renewed nomenclature of Vtx-producing *E. coli *is proposed to avoid more confusion in communication during future outbreaks and to replace the acronym EHEC that only refers to a clinical condition.

## Table of contents

1. Introduction

2. Verotoxin-producing and enteroaggregative *E. coli*

2.1. Verotoxin-producing *E. coli *with attaching and effacing properties

2.2. Verotoxin-producing *E. coli *without attaching and effacing properties

2.3. Enteroaggregative *E. coli*

3. The O104:H4 as compared to O157:H7 Verotoxin-producing *E. coli *outbreaks

3.1. Characteristics of the O104:H4 *E. coli *strain

3.2. Reservoirs and transmission

3.3. Clinical and epidemiological data

4. Virulence factors and host specificity of AE-VTEC and Agg-VTEC

4.1. The Verotoxins

4.2. The AE lesion

4.3. The AAF adhesins

5. General discussion

6. Competing interests

7. Authors' contributions

8. References

## Introduction

*Escherichia coli *is a well-known Gram-negative, rod-shaped bacterium belonging to the family *Enterobacteriaceae *that readily grows on simple bacteriological media. While many strains occur as commensal members of the microbiota in the intestinal tract of animals and humans, some strains are, however, important pathogens, that cause a wide spectrum of diseases, ranging from self-limiting to life-threatening intestinal and extra-intestinal illnesses, like enteritis, enterotoxaemia, cystitis, pyelonephritis, meningitis, mastitis, arthritis, and septicaemia [1-3].

Many pathogenic *E. coli *are host-adapted and only a limited number of strains infecting animals are able to cause disease in humans. These strains, however, are more likely to get the attention of the scientific community and of the news media. A typical example of zoonotic *E. coli *is the O157:H7 serotype that can be responsible for two severe syndromes in humans, haemorrhagic colitis (HC) and haemolytic uremic syndrome (HUS), and whose reservoir is the intestinal tract of healthy cattle and other ruminants [1,4,5]. The hallmark of these "enterohaemorrhagic *E. coli*" ("EHEC") strains is the production of cytotoxins called Vero(cyto)toxins (Vtx) or Shiga toxins (Stx), two synonyms for the same group of toxins referring either to their toxicity for Vero cells [6] or to their homology with the Shiga toxin produced by *Shigella dysenteriae *type 1 [7]. Another key property of O157:H7 "EHEC" strains is the production of the ultrastructural "Attaching and Effacing" (AE) lesion [1,4,5]. Although *E. coli *strains "possessing the same clinical, epidemiological and pathogenic properties as O157:H7 strains" in humans can also be designed as "enterohaemorrhagic *E. coli*" [8], the essential genetic features that define organisms capable of causing either HC or HUS clinical syndromes are not clear [3,9].

In May 2011, the Robert Koch Institute (Berlin, Germany) issued an alert on an increase of the number of cases of HC and HUS observed in Germany. Though such clinical signs are often associated with "EHEC" infections, laboratory investigations pointed out that this outbreak was caused by a strain of serotype O104:H4 combining the adherence properties of enteroaggregative *E. coli *(EAggEC) and the production of Vtx, but negative for the AE lesion; therefore not corresponding to the above definition of "EHEC". Nevertheless the O104:H4 strain was often described as an "EHEC bacterium" in the media, on different internet sites and in scientific publications [10,11], adding to the confusion and misleading information during the outbreak, a.o. about the actual origin of the bacterium, since "EHEC" outbreaks are most often of animal origin while no animal reservoir has ever been identified for EAggEC [1,4,5].

The purpose of this manuscript is therefore to explain the actual situation to the medical and veterinarian professions by (i) comparing the epidemiology and clinical data of the O104:H4 strain outbreak with the classical O157:H7 "EHEC" outbreaks; and (ii) describing the most important virulence properties of Vtx-producing *E. coli *(VTEC) and of EAggEC, with emphasis on those involved in host specificity. In a final section, an adapted nomenclature of VTEC is proposed to avoid more confusion in the future if strains combining Vtx-production with other virulence factors are described. But at first the different VTEC and EAggEC strains will be briefly described.

### Verotoxin-producing and enteroaggregative *E. coli*

The pathogenic strains of *E. coli *produce specific virulence factors that facilitate their interactions with the target host: colonization of the epithelial surfaces, crossing of the mucosal barriers, invasion of the blood stream and internal organs, and/or production of toxins causing cellular and tissue damages leading to organ dysfunction, clinical signs, symptoms and diseases. They are grouped in so-called "pathotypes" (or "virulotypes") on the basis of four criteria: the clinical syndrome, the target species, the adherence factors, and/or the production of exotoxins (Table 1). Several review articles on the *E. coli *pathotypes in humans and animals have been published [1,2,4,12,13]. Only the VTEC and EAggEC pathotypes of interest for this manuscript are described in some details hereunder.

**Table 1:** Definition, host range and virulence properties of enteric and enterotoxaemic *E. coli *in animals and humans (from [1,2,4,12-16])

Name (Acronym) *	Target host range	Diseases	Virulence	Remarks
Enteroinvasive (EIEC)	Humans, primates	Dysentery	Invasion of and multiplication in the enterocytes	Similar to *Shigella sp*.
Enterotoxigenic (ETEC)	Pigs, ruminants, humans (more rarely dogs)	Traveller's diarrhoea; profuse neonatal diarrhoea in babies, calves and piglets; post-weaning diarrhoea in piglets	Fimbrial adhesins (F2 to F6, F17, F18, F41, ...); heat-stable (STa, STb) and heat-labile (LT1, LT2) enterotoxins	
Enteropathogenic (EPEC)	Humans, all mammals	Diarrhoea	Attaching and effacing (AE) lesion; type 4 BFP fimbriae by typical (t) EPEC of humans	Localized adherence (LA) of tEPEC on cells in culture; LA-like adherence of atypical (a) EPEC at the cell surface
Verotoxigenic or Shigatoxigenic (VTEC or STEC) *	Humans, piglets	Haemolytic-uremic syndrome (HUS) in humans; oedema disease in piglets	Verotoxins (Vtx); afimbrial (Saa by human or AIDA by porcine VTEC) and fimbrial (F18 by porcine VTEC) adhesins	Ruminants can be healthy carriers of human VTEC (= reservoir host)
Enterohaemorrhagic (EHEC) *	Humans	(haemorrhagic) colitis and HUS in humans; diarrhoea in young calves	Vtx and AE lesion	Ruminants can be healthy carriers of human EHEC (= reservoir host)
Enteroadherent or Enteroaggregative (EAEC or EAggEC)	Humans (sporadically in animals)	Diarrhoea	Human EAggEC: small fimbrial adhesins (AAF/Hda); toxins (Pet, EAST1, ShET1); transcriptional activator gene (*aggR*)	Aggregative "stacked brick" adherence (AggA) on cells in culture; animal EAggEC are different from human EAggEC
Diffusely adherent (DAEC)	Humans, animals	Diarrhoea; extra-intestinal infections (urinary tract infections, septicaemia)	Diffuse adherence (DA) on cell culture mediated by adhesins of the AFimbrial Adhesin (AFA) family, or by AIDA adhesin	
Necrotoxigenic (NTEC)	Humans, animals (NTEC1); ruminants (NTEC2)	Diarrhoea; extra-intestinal infections (urinary tract infections, septicaemia)	Cytotoxic Necrotizing Factors (CNF) 1 or 2; different fimbrial (Pap, Sfa and/or F17) and/or afimbrial adhesins (AFA family)	

* see Introduction and Section 2.1 for the definitions of the names and acronyms of pathogenic *E. coli *strains.

### Verotoxin-producing *E. coli *with attaching and effacing properties

**Figure 1:**
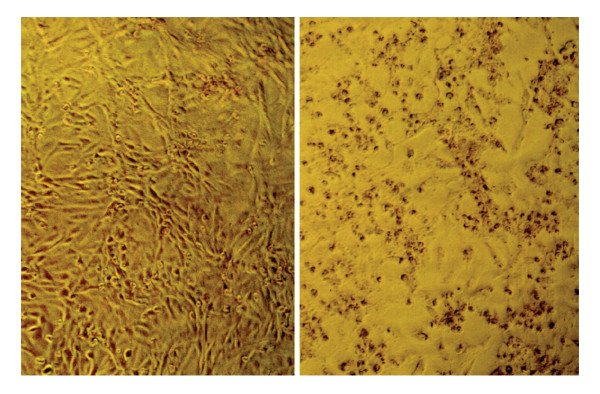
**Cytotoxic effect of Verotoxin (Vtx) 1 produced by an O157:H7 AE-VTEC**. Untreated (left) and treated (right) Vero cells in culture (DP collection).

**Figure 2:**
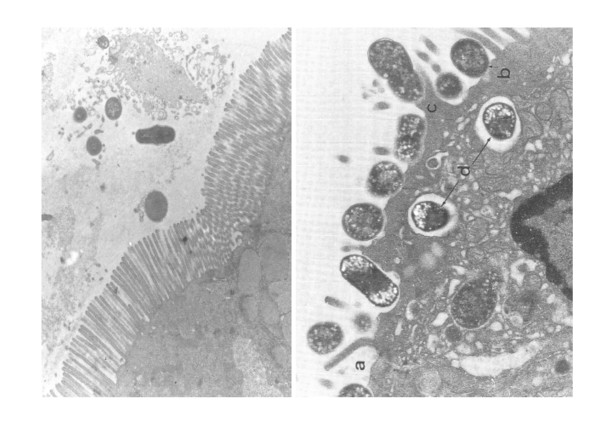
**Attaching and effacing (AE) lesions produced by a canine enteropathogenic *E. coli *(EPEC) in the ligated loop intestinal assay in rabbit**. Healthy enterocyte with microvillus layer (left); AE lesion (right) from [20]: a) few "surviving" microvilli; b) intimately adherent *E. coli*; c) pedestal formation beneath the adherent bacteria; d) sometimes internalization of the bacteria into the enterocytes.

VTEC are defined by the production of cytotoxins killing Vero and some other (HeLa, HEp-II, MDBK) cell lines (Figure 1), designated by two synonymous names, Verotoxins (Vtx) or Shiga toxins (Stx). The acronym "EHEC" was proposed by Nataro and Kaper [4] for all VTEC, distinguishing "typical EHEC" that produce the AE lesion on enterocytes (Figure 2) and "atypical EHEC" that do not. The same authors later considered that "EHEC" are a subgroup of only AE-positive VTEC [1]. But not all authors actually agree on the definition of this acronym "EHEC" [1,2,4,12,13] and, as a consequence, the range of *E. coli *strains covered by this acronym varies in the scientific literature. Moreover AE lesions are also produced by enteropathogenic *E. coli *(EPEC) that produce no Vtx [14,17,18] (Table 1) and some authors still group together AE lesion-producing VTEC and EPEC under the name "Attaching Effacing *E. coli*" (acronym AEEC) [19] adding to the confusion of the nomenclature. Therefore to avoid any confusion in this manuscript the acronym "EHEC" will be disregarded and replaced by the acronym "AE-VTEC".

AE-VTEC are responsible for uncomplicated diarrhoea and HC in humans, sometimes progressing to HUS or in adults to thrombotic-thrombocytopenic purpura (TTP). Both HUS and TTP syndromes include microangiopathic anaemia, thrombocytopenia, renal failure and often central nervous system involvement [5,21]. In summary the AE lesion is responsible for the non-haemorrhagic enteritis, while the Vtx are responsible for HC, HUS and TTP. AE-VTEC strains belonging to serotype O157:H7 have the highest epidemiological and clinical incidence and are the most frequently involved in severe and large outbreaks (seropathotype group A) [22]. Other clinically important AE-VTEC serotypes, also causing severe diseases in humans and involved in outbreaks though less frequently, are O26:H11, O103:H2, O111:H-, O121:H19, and O145:H- (seropathotype group B) [22].

AE-VTEC are primarily human pathogens, but they are also of interest for veterinarians because their main reservoir hosts are the ruminants, including cattle, sheep, goats, and cervids, which are asymptomatic carriers. In many cases, human infection occurs via consumption of vegetal and animal foodstuffs contaminated by the faeces of ruminants (mainly cattle). Moreover AE-VTEC of serotypes O5:H-, O26:H11, O111:H- and O118:H16 can also cause diarrhoea in less than 3 month-old calves [1,4,12,14,21,23-25]. Besides the production of Vtx and of AE lesions, other virulence properties of AE-VTEC strains have not been formally identified yet, though many candidates have been proposed more especially regarding colonization factors of the human and ruminant intestines [5,24,25].

### Verotoxin-producing *E. coli *without attaching and effacing properties

AE-negative VTEC (that we will further designated as VTEC without suffix unless specific adherence factors are known) can be subdivided into porcine, human and ruminant strains. The porcine VTEC responsible for the oedema disease syndrome in weaned piglets form a target host specific group of strains belonging to specific serotypes (like O138:K81, O139:K82, O141:K85) and producing the Vtx2e variant (subtyping nomenclature established at the 7th International Symposium on Shiga Toxin < Verocytotoxin > -Producing *Escherichia coli *Infections, Buenos Aires, 10 to 13 May 2009 [26]) and the F18 target host specific adherence factor allowing the colonization of the porcine intestine [2,27]. These F18-VTEC will not be discussed in this manuscript.

When infecting humans, VTEC can also be responsible for HUS due to the production of Vtx. The clinically and epidemiologically most important human VTEC, though they only sporadically cause outbreaks, belong to the O91:H21, O104:H21, and O113:H21 serotypes [22] (seropathotype group C). Such VTEC can also produce several additional virulence factors, including different adhesins that can be involved in intestinal colonization of humans [28,29]. In addition other VTEC serotypes, whose epidemiological role is currently unknown, have been sporadically associated with either mild or no symptoms in humans so far [22] (seropathotype group D). Finally some VTEC serotypes have never been isolated from humans [22] (seropathotype group E). All seropathotypes of VTEC can be isolated from faeces of asymptomatic ruminant carriers [28,30].

### Enteroaggregative *E. coli*

**Figure 3:**
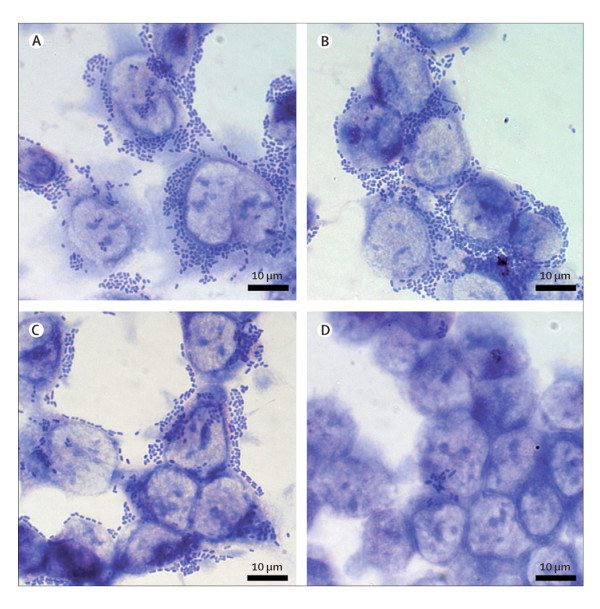
**Aggregative "stacked-brick" adherence to cultured intestinal epithelial cells**. Outbreak *E. coli *O104:H4 isolate LB226692 (A); an enteroaggregative *E. coli *(EAggEC) strain (positive control) (B); HUSEC041 (C); HUSEC037 (negative control) (D). Reproduced from [33] with permission (licence number 2775450359431, October 24, 2011).

The acronym EAggEC refers to *E. coli *strains characterized by a specific "stacked brick" aggregative adherence pattern on Hep-II [31,32], a cell line of human origin (Figure 3). EAggEC represent emerging specific human pathogens responsible for acute or persistent diarrhoea in children and adults worldwide. EAggEC strains produce specific adhesins (Aggregative Adherence Fimbriae or AAF) responsible for the adherence on Hep-II cells and for the intestinal colonization. But EAggEC are a heterogeneous group of strains in their properties and whole virulence gene repertoires and not all seem to be pathogens.

Dozens of EAggEC O:H serotypes have indeed been described so far, but many EAggEC can not be typed, because either they auto-agglutinate due to the production of AAF adhesins or they produce still unidentified O antigens. Moreover EAggEC can produce different combinations of three toxins (the plasmid-encoded autotransporter toxin Pet, the enteroaggregative heat-stable toxin EAST1, and the *Shigella *enterotoxin ShET1). The actual role of these toxins in pathogenesis is however unclear, since they can also be produced by other pathogenic *E. coli *strains. Today the highest correlation between the aggregative adherence phenotype on cell culture and diarrhoea in humans is the presence of the *aggR *gene coding for a transcriptional regulator of the expression of several virulence-associated genes [34-37].

### The O104:H4 as compared to O157:H7 Verotoxin-producing *E. coli *outbreaks

#### Characteristics of the O104:H4 *E. coli *strain

Although HC and HUS that are often observed during AE-VTEC infections were reported as main clinical signs during the German outbreak, the O104:H4 *E. coli *strain isolated from patient stools was positive for the gene coding for Vtx2a (subtyping nomenclature established at the 7th International Symposium on Shiga Toxin < Verocytotoxin > -Producing *Escherichia coli *Infections, Buenos Aires, 10 to 13 May 2009 [26]), and negative for genes coding for the AE lesion [33,38]. Further genetic analysis of this O104:H4 VTEC strain identified additional pathogenic determinants, some typical of VTEC like the *ter *gene cluster conferring tellurite resistance, but also others typical of EAggEC like the *aggR *gene regulator, the *aafA *gene encoding the AAF/I adhesin and the *set1 *gene encoding the ShET1 toxin. Conversely the *ast1 *gene encoding the EAST1 toxin was absent [33,38]. The O104:H4 outbreak strain is therefore a chimeric pathogenic *E. coli *here designated as Agg-VTEC i.e. an enteroaggregative Vtx-producing *E. coli*.

Such Agg-VTEC strains of serotype O104:H4 were already isolated in Germany in two HUS cases in 2001, but differed by some virulence factors, for instance the presence of the *ast1 *gene [33]. Agg-VTEC have been rarely described and they belonged to other serotypes, like O111:H2 [39] and O86:H- [40]. Another unique characteristic of the 2011 German O104:H4 Agg-VTEC outbreak strain was the production of a CTX-M15 extended-spectrum β-lactamase (ESBL) [33]. On this basis, several enrichment and selective agar plates were used during the investigation of the outbreak, followed by agglutination or molecular techniques for *vtx, wzx_O104 _*and/or *fliC_H4 _*genes [33,38,41]. However, selective media containing antibiotics should be used cautiously, as not only O104:H4 strains isolated in Germany in 2001 do not possess, but also derivatives of the 2011 outbreak strain lost the ESBL-encoding genes [33,42].

### Reservoirs and transmission

In the first days of the outbreak, involvement of an AE-VTEC strain was reported and ruminants were logically suspected as source of contamination. Indeed, the primary sources and vehicles of O157:H7 AE-VTEC infections are ruminants, mainly cattle and sheep in which the bacteria are not pathogenic [1,4,12,14,21,23-25]. Contamination of humans occurs through very diverse vehicles after direct or indirect faecal contamination, like meat (generally undercooked ground beef), unpasteurised dairy products, fresh vegetables, fruit, drinking or swimming water and/or pet farms. Though secondary spread is also common, humans represent a dead-end target host of O157:H7 AE-VTEC and the source-sink epidemiological model can be used [23]. Shedding of O157:H7 AE-VTEC in children has a median duration of 13 days (range 2 to 62 days) in patients with diarrhoea or HC [43] and 21 days (range from 5 to 124 days) in patients with HUS [43]. By contrast with *Salmonella enterica *serotype Typhi no long-term carriage has been described [23].

Identification of the O104:H4 strain as an Agg-VTEC radically modified the epidemiological perspective and investigations. Indeed EAggEC are present in all human populations all over the world but no animal reservoir has been identified so far. *E. coli *strains displaying the aggregative adherence pattern on cells in culture were isolated from faeces of calves, piglets and horses suffering diarrhoea in South America, but they were unrelated to human EAggEC strains in their major phenotypic traits and in their virulence genes, being negative a.o. for the *aggR *and *aaf *genes [15,44,45]. In addition to being a frequent cause of diarrhoea in children or adults worldwide EAggEC have already been implicated in food-borne diarrheic outbreaks in developed and developing countries after human-borne contamination during food handling [4,34-37,46,47].

No data are currently available on the actual reservoir of Agg-VTEC strains [38] but all epidemiological data point to humans and the faecal-oral route as the most probable primary source of contamination during the German outbreak. The first case-control studies pointed to fresh vegetables, leafy salad, tomatoes and cucumbers as potential vehicles [48]. However new epidemiological investigations identified germinated sprouts as the infection source [49-51] though no bacteriological confirmation has been issued until now. Further epidemiological data traced the source of infection back to a lot of fenugreek seeds used by the producer of the germinated sprouts in Germany and originating from Egypt as the most probable origin of the O104:H4 Agg-VTEC strain [49].

Fresh produce is increasingly recognized as vehicle of food-borne pathogens and are often consumed as mixed food products, making it difficult to identify the contamination source [52]. Sprouted seeds were involved in multiple *Salmonella enterica *and O157:H7 AE-VTEC infections [53]. In particular, the massive outbreak of O157:H7 AE-VTEC in schoolchildren in 1996 in Sakai, Japan, was undoubtedly related to contaminated radish sprouts, although bacteriological tests on food and seeds remained all negative [54]. In their review on fresh fruit and vegetables as vehicle for the transmission of human pathogens, Berger et al. [52] emphasize an increasing risk due to proliferation of pathogens during the processing and post-harvest handling procedures of contaminated seeds.

### Clinical and epidemiological data

At the end of the outbreak, the Robert Koch Institute [55] reported 3979 probable and confirmed cases including 938 HUS cases and 3041 non-HUS cases (Table 2). A total of 55 infected persons died, corresponding to 1.4% of the reported cases. The case fatality rate was much higher in HUS cases (3.7%) than in non-HUS cases (0.54%). Most cases were recorded in Germany (3842 cases, 96.7%). The majority of patients from 14 other European countries, USA and Canada, reported travel to or contact with travellers to Germany. A notable exception are the 15 HUS cases during an event in Bègles, a village in the region of Bordeaux, France, with no direct or indirect contact with Germany, but who also consumed germinated sprouts made from the same lot of fenugreek seeds from Egypt, increasing the likelihood that these fenugreek seeds are the actual original source of infection [56]. Furthermore, Pulsed Field Gel Electrophoresis (PFGE) analysis of German and French isolates showed identical profiles between all investigated isolates [57].

**Table 2:** Epidemiological and clinical characteristics of O104:H4 Agg-VTEC infections as compared to O157:H7 AE-VTEC infections

	AE-VTEC O157:H7	Agg-VTEC O104:H4
Incidence of sporadic cases	Third bacterial cause of diarrhoea in developed countries	Extremely rarely reported
Outbreaks	Frequent (from a few to > 6000 cases)	One outbreak (almost 4000 cases)
Age distribution	Young children (elderly)	Middle aged adults
Gender distribution	Equal	Predominance of female
Incubation	1 to 9 days (average 3-5 days)	8 days (interquartile range 7 to 9 days)
Progression to HUS	7-10%	> 20%
Mortality	0.5%	1.4%

The severity of illness was markedly higher during the O104:H4 Agg-VTEC outbreak with more than 20% of HUS cases and 1.4% deaths as compared to average figures during O157:H7 AE-VTEC outbreaks with 7-10% HUS cases affecting predominantly young children and 0.5% overall death rate (Table 2) [58]. Possible explanations are (i) the involvement of an Agg-VTEC strain that is a human-adapted pathogen and maybe a better gut colonizer than O157:H7 AE-VTEC and (ii) the use of antibiotics during the outbreak. Indeed though they are generally contra-indicated during VTEC infections [21], as toxin production can be induced by some of them (like fluoroquinolones) [59], it can be assumed that antibiotics are commonly used during hospitalization for HC. Since the O104:H4 Agg-VTEC strain produces an ESBL, use of broad-spectrum beta-lactams might have contributed to the outgrowth of this strain in the gut.

The German outbreak by the O104:H4 Agg-VTEC differed from the classical O157:H7 AE-VTEC outbreaks by several other epidemiological characteristics (Table 2) [58]. The incidence of O157:H7 AE-VTEC is not negligible since 350 outbreaks were reported in the USA between 1982 and 2002 and many sporadic cases were recorded as well [21]. According to ECDC, the overall notification rate in Europe was 0.66 cases per 100, 000 inhabitants in 2008 and remained unchanged over the last few years [60]. By contrast, only a few sporadic cases related to VTEC of serotype O104:H4 were reported before the German outbreak and even less isolates were identified as EAggEC [38]. This massive outbreak and emergence of an Agg-VTEC of serotype O104:H4 was therefore totally unexpected. The age and gender distribution also represents a distinct contrast with O157:H7 AE-VTEC outbreaks: most affected cases were middle-aged adults (median age was 43 years) with a predominance of women (68% of HUS and 58% of non-HUS cases), whereas during O157:H7 AE-VTEC outbreaks HUS occurs mainly in young children and in elderly people equally in both sex [55]. This particular age and gender distribution may reflect either relative efficacy of the human gut colonization by this Agg-VTEC strain or, more probably, dietary preferences, as already observed during a particular spinach-associated O157:H7 AE-VTEC outbreak in the United States in 2006. Here too, the majority of HUS patients were adult women in contrast to the classical age and gender distribution during O157:H7 AE-VTEC infections [61].

### Virulence factors and host specificity of AE-VTEC and Agg-VTEC

Whole genome sequence analysis of VTEC sensu *lato *and EAggEC identified numerous genes coding for putative adherence factors to colonize the intestinal epithelium and/or toxins to cause lesions on the host cells [24,28,29,34-37]. Some of them most probably also represent a basis of their host specificity either in disease (target host) or in carrier state (reservoir host). Nevertheless, the actual roles in disease of only the Verotoxins of VTEC sensu *lato*, the AE lesion of AE-VTEC, and the AAF fimbrial adhesins of EAggEC have been extensively studied.

### The Verotoxins

In 1977, Konowalchuk et al. observed in vitro that culture supernatants of some *E. coli *isolates produced a profound cytotoxic effect on Vero cells [6]. The clinical significance of Verotoxins (Vtx) remained obscure until 1983, when their association with HC and HUS was established [62,63]. Two distinct Vtx families have been described, Vtx1 and Vtx2. Vtx1 is nearly identical to the Shiga toxin of *Shigella dysenteriae *serotype I and the term Shiga toxin (Stx) was later proposed to design this group of cytotoxins [7]. No consensus has been reached in the scientific community and both terms can be used interchangeably. According to the subtyping nomenclature established at the 7th International Symposium on Shiga Toxin < Verocytotoxin > -Producing *Escherichia coli *Infections, Buenos Aires, 10 to 13 May 2009 [26], three variant forms of Vtx1 and seven variant forms of Vtx2 have been described, called Vtx1a, Vtx1c & Vtx1d and Vtx2a through Vtx2g. Strains producing Vtx2a, Vtx2c and/or Vtx2d are more often associated with HUS [23,64]. Vtx2e is associated with the oedema disease in swine and is only exceptionally produced by human isolates [2,65]. The majority of Vtx are encoded by phage-located genes that can be lost or acquired by other *E. coli *not only in vitro but also in vivo [66-68].

Vtx are dimeric toxins comprising one A subunit, responsible for the enzymatic activity and five B subunits that bind to a cell surface receptor, Gb3 (Gb4 for Vtx2e). In humans the Gb3 receptor is expressed at low level by enterocytes and at high level by endothelial cells in particular in the renal glomeruli. Vtx cross the intestinal epithelium by transcytosis through the enterocytes, i.e. endocytosis followed exocytosis at the basal pole. Vtx subsequently travel in the blood stream in association with leucocytes and attach to receptors on the endothelial cells. After internalization, the whole toxin is transported retrogradely within endocytic vesicles to the endoplasmic reticulum via the Golgi apparatus. There the A subunit is translocated into the cytoplasm, enzymatically activated and reaches its target, the 28S ribosomal RNA. The protein synthesis is inhibited after N-glycosylation of a specific adenine residue of the 28S rRNA. By damaging the blood vessel wall, Vtx eventually provoke thrombotic microangiopathy, leading to the three prominent features of HUS, haemolysis, thrombocytopaenia and renal failure [12,21,24,69,70].

Though a few AE-VTEC serotypes (O5:H-, O26:H11, O111:H-, O118:H16) can also cause diarrhoea in less than 3 month-old calves and association of O157:H7 AE-VTEC with neonatal diarrhoea in calves has been sporadically reported, there exists no report of HUS-like syndrome in ruminants [2,12,14,21,23,24]. It has long been hypothesised that the absence of receptors on enterocytes [71] was the cause of this lack of Vtx toxicity in cattle, but Gb3 has been recently detected on bovine intestinal and renal cells [72]. A different intracellular trafficking in ruminants localizes Vtx in lysosomes, leading to abrogation of transcytosis in enterocytes [73].

### The AE lesion

Another important characteristic of AE-VTEC strains is their ability to provoke the disappearance of the enterocyte microvilli and to intimately attach to their nude cytoplasmic membrane (Figure 2). AE lesions are the results of a profound dis- and re-organization of the actin filaments of the eukaryotic cell skeleton [17,18,21]. As mentioned earlier, AE lesions are also induced by EPEC strains that produce no Vtx [14,17,18] (Table 1).

A large bacterial chromosomal pathogenicity island, the locus of enterocyte effacement (LEE), carries all (or almost all according to the sero-pathotypes) genes necessary for the formation of the AE lesion [17,18,21]. They code for the proteins building up a type 3 secretion system (T3SS) or needle-like injectisome apparatus, for type 3-secreted proteins or translocated effectors (whose repertoires can be different according to the seropathotypes) interacting through a phosphorylation and activation cascade with the cell cytoskeleton actin filaments leading to the effacement of the enterocyte microvilli, and for the intimin adhesin that is a bacterial outer membrane protein responsible for the intimate (< 10 nm gap) attachment of the *E. coli *to the enterocytes. The injectisome tip is embedded within the enterocyte cytoplasmic membrane forming a bridge between the bacteria and the enterocytes, through which the effectors translocate into the enterocyte. One of them is the Tir (Translocated Intimin Receptor) protein that can have different functions after incorporation into the enterocyte cytoplasmic membrane, including serving as a receptor for the intimin adhesin [17,18,21].

Different types of intimin adhesins have been described that differ in their amino acid carboxy-terminal sequences. These intimins are designated by Greek letters and correlated to the AE-VTEC and EPEC serotypes [18,74]. The intimin adhesin of O157:H7 AE-VTEC strains belongs to the gamma type (Int-γ). Since the interaction between intimin and Tir is located in the carboxy-terminal region of the intimin, the different intimin types specifically interact with their cognate Tir receptor [75,76]. Nevertheless this specific molecular interaction does not fully explain host specificity and tissue tropism.

The reservoir (ruminants) and target (humans) host specificity and tissue tropism (jejunum, colon, rectum) might therefore reside in the production of non-intimin adhesins acting as primary colonization factors. Several have been identified and their role studied, more especially on cells in culture [28]. In addition whole genome sequencing of different AE-VTEC strains (especially O157:H7) [77-80] revealed the presence of genes coding for several putative fimbrial and non-fimbrial adhesins [81]. But as a matter of fact the actual role of these (putative) adhesins in the pathogenicity, host specificity and tissue tropism of AE-VTEC still awaits full characterization.

### The AAF adhesins

The aggregative adherence phenotype of human EAggEC strains to HEp-2 or HeLa cells in culture [31,32] is associated with the production of four types of fimbrial adhesins: AAF (Aggregative Adherence Fimbriae)/I, AAF/II, AAF/III or Hda (HUS-associated Diffuse Adherence), whose encoding genes (*aaf *and *hda*) are plasmid-located [82-85]. If the three AAF adhesins are EAggEC-specific, interestingly the *hda *gene is also present in HUS-associated *E. coli *as its name indicates [83]. AAF fimbriae are long thin and flexible fimbrial appendices of 2 to 3 nm in diameter that are genetically distantly related to the large Afimbrial Adhesin (AFA) family [86].

In vivo EAggEC not only adhere to enterocytes and to the extracellular intestinal matrix, but also form mucoid biofilms at the surface of the intestinal epithelium of the terminal ileum and of the colon [37,87,88]. However the precise mechanism by which EAggEC cause diarrhoea is still not fully understood. Several putative additional virulence factors, including different toxins as already mentioned, have been identified but none has yet been proved as the actual cause of diarrhoea [37,87,89]. EAggEC actually form a heterogeneous group of strains harbouring different combinations of those putative virulence genes, some of them being regarded as pathogens while others not [34-37].

The highest correlation between this phenotype on cell culture and diarrhoea in humans is the presence of the *aggR *gene located on the same plasmid as the *aaf *genes. AggR is a transcriptional activator regulating the expression of several virulence-associated genes, including the *aaf *genes [34-37]. Host specificity of EAggEC for humans is most probably based on the AAF fimbrial adhesins since all EAggEC isolated so far from animals harbour no *aaf *gene [15].

## General discussion

Many virulence-associated properties of pathogenic *E. coli *are encoded by genes located on mobile genetic elements, like plasmids, phages, transposons, and pathogenicity islands. For instance, it has been observed that Vtx-encoding phages can be acquired, or lost in vitro and in vivo. Therefore in theory new combinations of virulence factors and VTEC pathotypes may arise anytime [66-68], rendering their nomenclature most difficult and confusing. It is today accepted that the prototype O157:H7 AE-VTEC strain originated from an O55:H7 EPEC strain by stepwise acquisition of VT-encoding phages followed by acquisition or loss of other properties [90,91] while AE-VTEC strains belonging to other serotypes followed other evolutionary lineages [9,80,92,93]. Analysis of the complete genome of the O104:H4 Agg-VTEC outbreak strain and of 7 Vtx-negative O104:H4 EAggEC strains actually confirmed that the outbreak strain is derived from the latter strains by horizontal transfer of Vtx-encoding genes [10,59,93].

Although one of them has until now strongly advocated the distinction between EHEC, VTEC and EPEC on the basis of the combination of Vtx and/or AE lesion production [27], today the authors of this review manuscript propose to change the nomenclature of Vtx-producing *E. coli*. At first they suggest to only use the acronym VTEC that refers to the production of Vtx and to disregard the "EHEC" and related (atypical EHEC, EHEC-like, EAHEC) acronyms [93] that refer to a clinical condition. "EHEC" is even further confusing since bloody diarrhoea is not always prominently present during clinical manifestation and since this acronym is actually used with various definitions in the scientific and medical literature. The second step in the identification would be to add a suffix to the acronym VTEC in order to better describe the other virulence-associated properties, more especially adherence properties [29]: AE-VTEC for those producing AE lesion (like O157:H7 and other "EHEC" serotypes), Agg-VTEC for those producing AAF/Hda adhesins (like the O104:H4), Saa-VTEC for those producing the Saa afimbrial adhesin, F18-VTEC for those associated with oedema disease in piglets, etc. Such nomenclature not only focuses on the main virulence-associated properties of VTEC, but also leaves the options open to include new combinations that might arise in the future. Moreover these acronyms can easily be included in the seropathotype grouping scheme of Karmali et al. [22]: for instance strains of seropathotype groups A and B are AE-VTEC; those of group C are either AE-VTEC or Saa-VTEC or the newcomer O104:H4 Agg-VTEC, etc. In addition, the communication about source and vehicle of contamination by veterinarians, medical doctors, scientists, journalists and politicians to the general public would be facilitated since an outbreak caused by an AE-VTEC strain would most probably find its origin in ruminant faeces, while an outbreak by another type of VTEC less probably.

Finally, some questions remain unanswered about the O104:H4 Agg-VTEC outbreak. The high proportion of adult patients developing HUS as compared with the young children predominantly infected with O157:H7 AE-VTEC, suggests that the Vtx2a crossed the intestinal mucosa more efficiently and/or in higher amounts, maybe as a consequence of the colonization of different intestinal segment(s) by the O104:H4 Agg-VTEC, although the actual reason remains unidentified. And the particular gender and age distribution also suggests that some dietary preferences could be a crucial factor. Another issue is the lack of investigations in Egypt. If it is confirmed that the fenugreek seeds are the origin of infection, it would be appealing to investigate how they were contaminated. Further, the survival and multiplication of the bacteria during sprouting and the means how to control the bacterial proliferation should be studied.

## Competing interests

The authors declare that they have no competing interests.

## Authors' contributions

All authors drafted different sections of the manuscript. JM and DP coordinated the work of the first and of the revised versions, respectively. HDG and FH critically read and revised the coordinated manuscripts. All authors approved the final manuscript.
